# Depressive disorders after mild craniocerebral injuries in amateur athletes

**DOI:** 10.1192/j.eurpsy.2021.1841

**Published:** 2021-08-13

**Authors:** N. Syrmos, G. Ampatzidis

**Affiliations:** Aristotle University Of Thessaloniki, Thessaloniki, Greece

**Keywords:** Depressive disorders, craniocerebral injuries, athletes

## Abstract

**Introduction:**

Craniocerebral injuries are serious traumatic situations

**Objectives:**

Aim of this study is to present cases of deppresive disorders after mild craniocerebral injuries in amateur athletes

**Methods:**

10 cases are presented. Range of age between 20 and 40 years old. All of them reported depressive disorders during the post traumatic period after mild craniocerebral injuries mainly during amateur athletic activities

**Results:**

All of them they receive appropriate neurological, psychiatric, psycological and rehabilitation support and treatment. They managed to have a good outcome after 12 months follow up.

**Conclusions:**

The development of depressive disorders after such traumatic events remains a strong predictor of a variety of difunctions (social, personal, work etc). The emergence of depressive disorders in many cases remains unexplored and poorly understood. The effect into the the overall health remains a very important factor to investigate. The combination and collaboration of the various medical disciplines is essential in order to help young people.

**Disclosure:**

No significant relationships.

